# Modulation of Human β-Defensin-1 Production by Viruses

**DOI:** 10.3390/v9060153

**Published:** 2017-06-21

**Authors:** Lisa Kathleen Ryan, Gill Diamond

**Affiliations:** 1 University of Florida College of Medicine, Division of Infectious Disease, Department of Medicine and Global Medicine, 1600 SW Archer Road, Box 100277, Gainesville, FL 32610, USA; 2 University of Florida College of Dentistry, Department of Oral Biology, 1600 SW Archer Road, Box 100424, Gainesville, FL 32610, USA; gdiamond@dental.ufl.edu

**Keywords:** hBD-1, *DEFB1*, antimicrobial peptide, virus, monocytes, plasmacytoid dendritic cells, innate immunity, epithelial cells, defensin, gene regulation

## Abstract

While initially identified as a broad-spectrum antimicrobial peptide, constitutively expressed in epithelia, human β-defensin (hBD)-1 is now recognized to have a more complex pattern of expression of its gene, *DEFB1*, as well as activities that extend beyond direct antimicrobial. These observations suggest a complex role for hBD-1 in the host defense against viral infections, as evidenced by its expression in cells involved in viral defense, and its gene regulation in response to viral challenge. This regulation is observed both in vitro and in vivo in humans, as well as with the murine homolog, mBD-1. While numerous reviews have summarized the existing literature on β-defensin gene expression and activity, here we provide a focused review of relevant studies on the virus-mediated regulation of hBD-1 and how this regulation can provide a crucial aspect of the innate immune defense against viral infection.

## 1. Introduction

Human defensins are antimicrobial peptides endogenously produced by various cell types of the body. They have broad antimicrobial activity, killing a wide spectrum of microorganisms, including bacteria, viruses and fungi. The killing mechanisms of defensins against microbes are reviewed elsewhere [1,2]. Paradoxically, the induction of defensins is perpetrated by the very microorganisms that they kill and this induction is under tight genetic control, both by the innate immune system and in the defensin genes, their processing enzymes and their genes.

Endogenous production of defensins is one of the first lines of defense in innate immunity against viruses. There are three classes of defensins: α, β and θ. Each class has its own separate structure, cell types that produce defensins and mode of induction and processing. Only β-defensins are produced consistently across the vertebrate species, but are evolutionarily derived from an ancestral defensin, termed “big defensin” [1,3,4]. Studies focusing on the modulation of defensins by viruses to date and gaps in research will be the focus of this article.

## 2. Human β-Defensin-1

Human β-defensin (hBD)-1 is an antimicrobial peptide existing in multiple forms ranging from 36–47 amino acids (aa) and is encoded by the gene, *DEFB1* [5]. The forms found depended on where in the body they were isolated. In blood plasma, Valore et al. found the multiple forms of hBD-1 (36–47 aa) to be bound to carrier molecules that released each peptide form under acidic conditions. Using in situ hybridization, they localized the hBD-1 mRNA in the epithelial layers of the loops of Henle, distal tubules, and the collecting ducts of the kidney, the pancreas and the epithelial layers of the vagina, ectocervix, endocervix, uterus, and fallopian tubes in the female reproductive tract [6]. They also cloned the hBD-1 gene and located it within 150 kb of the α-defensin cluster on chromosome 8, band p23, thus showing that hBD-1 and the previously characterized neutrophil and Paneth cell defensins evolved from a common ancestral gene [7]. All forms of hBD-1 have antimicrobial activity but have variable activity under the conditions tested, such as varying osmolality [6] or redox state [8].

Classically, hBD-1 is known as the constitutively produced β-defensin in epithelial cells [9], produced mainly by epithelial cells in the kidney [6], lung [10], female reproductive tract [6], other mucosal organs [9] and skin [11]. It is thought to have a protective effect against invading organisms, aiding to provide a first-line innate immune defense against invaders [12]. However, this steady-state production of hBD-1 is not always the case, as hBD-1 is inducible under the right conditions. While *DEFB4* (which encodes hBD-2) and *DEFB103* (which encodes hBD-3) exhibit copy number variability, which can lead to variation in gene expression and peptide levels between individuals, hBD-1 does not appear to have such variability [13]. Thus, differences in basal and induced levels of expression are most likely due to other factors. The induction of hBD-1 is highly dependent on the cell type stimulated, the time of sampling after stimulation, the biomolecule or organism inducing it, whether the organism is living or not, and the environment and context of the cell being stimulated.

Recent research by our lab and others has demonstrated a much more complex role for hBD-1. There is evidence that hBD-1 is inducible by a different type of stimulus than the other hBDs and that individuals respond to different types of stimuli differently, suggesting a strong genetic component in this innate immune response to viruses and other microbes. The signaling pathway of induction of hBD-1 (expressed by *DEFB1*) is separate from that of hBD-2 and hBD-3 as well.

## 3. hBD-1 and hBD-2 Induction by Lipopolysaccharide

The first β-defensin that was characterized as inducible occurred in tracheal epithelial cells in cattle and was called tracheal antimicrobial peptide (TAP), induced by lipopolysaccharide (LPS) and the proinflammatory cytokines, tumor necrosis factor (TNF) and interleukin (IL)-1β via a nuclear factor- κB (NF-κB) pathway [14,15,16]. The human homologue then followed: LPS was shown to induce hBD-2 in airway epithelial cells [17]. Numerous other mucosal epithelial cells types, such as in the gastrointestinal tract, were also targets for LPS or proinflammatory cytokine induction of hBD-2 [18]. The induction of hBD-2 by LPS was shown to be mediated via Toll-like receptor (TLR) 4 and subsequent stimulation of an NF-κB pathway [19].

The first study to show the variability and inducibility of β-defensins in monocytic cells was a study by Duits et al. [20]. They studied hBD-1 and hBD-2 gene expression using semi-quantitative reverse transcription polymerase chain reaction (RT-PCR) in monocytes, monocyte-derived macrophages (MDM) and alveolar macrophages (AM) as well as monocyte-derived dendritic cells (MDDC) generated from monocytes using granulocyte-macrophage colony-stimulating factor (GM-CSF) and IL-4. The MDDC were matured into type 1 (using LPS + interferon (IFN)-γ) and type 2 (using LPS + prostaglandin E_2_) dendritic cells (DC), then the immature and mature DCs were analyzed. The cells were first isolated prior to stimulation using standard methods and all unstimulated cell types isolated expressed hBD-1 mRNA. Incubation with LPS and IFN-γ increased hBD-1 mRNA in a dose- and time-dependent manner in the monocytes and MDM, but AM continued to express the same level of hBD-1. Immature MDDC did not have much, if any, hBD-1 mRNA. However, as the MDDC matured into either type I or type II DCs, the expression of hBD-1 increased greatly. hBD-1 peptide was also detected in unstimulated monocytes and AM using immunohistochemistry. hBD-2 mRNA was undetectable in MDM, and at best only moderately expressed in monocytes, AM, and immature MDDC. hBD-2 increased in monocytes as they matured to MDM, but hBD-2 was not increased as the immature MDDC were matured to type 1 or type 2 DC. AM had only moderate levels of hBD-2 expression but LPS and IFN-γ greatly increased hBD-2 mRNA after 18 h. In all cases, the level of gene expression of both hBD-1 and hBD-2 varied among individuals.

Unlike the previous study, Fang et al. [21] did not detect constitutive hBD-1 or other hBDs in freshly isolated peripheral blood mononuclear cells (PBMC) using semi-quantitative RT-PCR. However, they performed the experiment differently. Instead of isolating the cells first, they incubated whole blood with 100 ng/mL LPS or with heat-killed *Pseudomonas aeruginosa* for 0, 3, 6, 12, and 24 h, then separated out the PBMC via Histopaque ^®^ (Sigma-Aldrich, St. Louis, MO, USA) density centrifugation and extracted total RNA and performed semi-quantitative RT-PCR. They observed transient mRNA production of hBD-1 and hBD-2 but not hBD-3. The mRNA level of hBD-1 and hBD-2 was detectable at 3 h following LPS or the bacterial stimulation, with a maximum at 6 h and a decrease at 12 h. The response of each individual to LPS in this study was variable. Some individuals did not respond at all. After induction with 100 ng/mL of LPS for 6 h, the vast majority (88.2%, 45 out of 51) of blood donors had detectable levels of mRNA for hBD-1 in the stimulated whole blood, whereas a relatively low rate of inducible expression in blood donors was found for hBD-2 (39.2%, 20 out of 51) [21].

Supporting the results of Duits et al. [20], we have detected small amounts of hBD-1 peptide via intracellular flow cytometry in unstimulated, freshly isolated PBMC and in gated CD11c^−^CD123^+^HLA-DR^+^ plasmacytoid dendritic cells (PDC), indicating that a small amount of peptide is present in these cells. In gated CD11c^+^CD123^−^HLA-DR^+^ myeloid-derived dendritic cells (MDC), no hBD-1 was detected in either stimulated or unstimulated cells. When the PBMC were isolated first, then stimulated with 100 mg/mL LPS and then gated for monocytes and PDC, hBD-1 levels increased. However, when ultrapurified LPS (containing no detectable bacterial protein contamination) was used to stimulate these cells, only monocytes had an increase in hBD-1. Thus, LPS was not a strong stimulator of PDC-derived hBD-1, but in monocytes, 100 ng/mL ultrapure LPS stimulated hBD-1 peptide in a dose- and time-dependent manner, appearing as early as 2 h and peaking at 6 h. Like the previous studies, the response to LPS varied from donor to donor in our study [22].

## 4. hBD-1 Induction by Viruses In Vitro

Our studies in monocytes and PDC infected with virus revealed that hBD-1 was inducible by both DNA and RNA enveloped viruses [23]. The magnitude of the hBD-1 response to virus was much greater than that of LPS and it varied greatly between individuals. Herpes simplex virus-1 (HSV-1), influenza A virus PR8 (H1N1), and Sendai virus incubated with either PBMC, purified PDC and purified monocytes stimulated the expression of hBD-1 mRNA and peptide, but not hBD-3 mRNA. hBD-2 was not induced by the viruses in monocytes until 18 h and not at all in PDC. The detection of hBD-1 peptide and mRNA was rapid; both mRNA and hBD-1 peptide was induced in as little as 2 h. Virus-stimulated IFN-α, in contrast, reached maximum levels in 6 h in PDC. When PDC were incubated with anti-IFN-α-receptor-1 antibody, no change in viral induction of hBD-1 by HSV-1 occurred [23,24].

Unlike in PDC stimulated with HSV-1, a study with M-tropic human immunodeficiency virus-1 (HIV-1) stimulated monocytes revealed that IFN-α responses are important for inducing hBD-1 following viral stimulation [25]. With HIV-1 incubation in vitro, they reported that PDC only showed a small increase of hBD-1 mRNA but monocytes significantly increased hBD-1 gene expression when infected with live HIV-1. Incubation with IFN-α also enhanced HIV-1-induced hBD-1 levels in monocytes, but TNF and IL-1β had no effect. hBD-1 in lymphocytes (CD3+ and CD19+ cells) and MDCs could not be induced by HIV-1.

In our studies of PDC [24], no increased hBD-1 gene expression occurred when cells were incubated with the TLR9 agonist, CpG-A, similar to the lack of a TLR9 agonist response with M-tropic HIV-1-infected monocytes [25]. Only live HSV-1 induced hBD-1 in PDC and this induction was blocked by an inhibitory CpG sequence, 5.5 µg/mL ODN2088 prior to infection. UV-inactivated HSV-1 did not induce hBD-1 [23]. In the HIV-1 patients’ monocytes, only 1–3% of the cells were permissive to viral entry, but viral entry was necessary to induce hBD-1 by HIV-1 [25]. These results indicate that the induction of hBD-1 is more complex and that hBD-1 is not induced exclusively via TLR9.

## 5. Transcriptional Control of hBD-1

**Figure 1:**
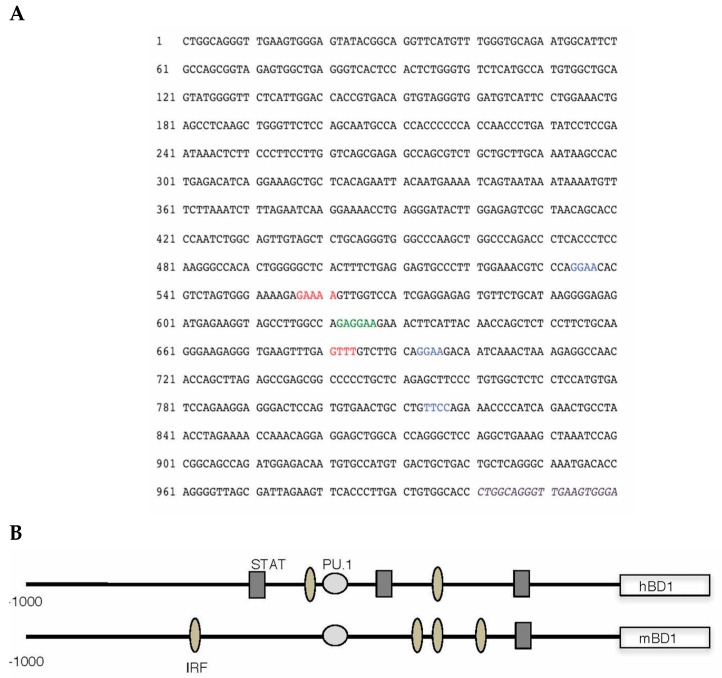
Genetic analysis of the regulatory element of the human β-defensin-1 (hBD-1) gene, *DEFB1*. The flanking sequence for human β-defensin-1 was obtained from GenBank (Accession no. U50930), and analyzed for putative transcription factor binding sites by computer using MatInspector software by Genomatix (Genomatix, GmbH, Munich, Germany). It is shown in sequence format in (**A**) and graphically in (**B**). Consensus sequence for PU.1/spleen focus forming virus (SFFV) proviral integration oncogene (SPI1) binding is shown in green (**A**) and as light gray circles (**B**). Interferon regulatory factor (IRF) consensus binding sites are blue (**A**) and oval shaded (**B**); Signal transducer and activator of transcription 1 (STAT1) consensus sequences are red (**A**) and gray rectangles (**B**). Sequence of exon 1 is in italics and purple.

**Figure 2:**
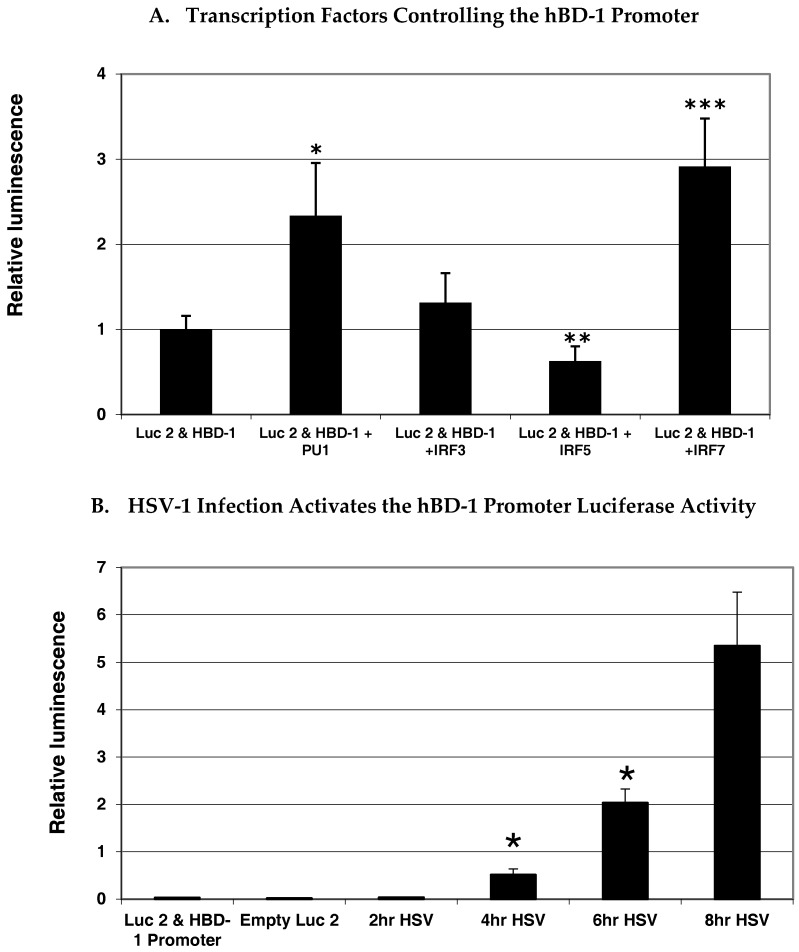
Transcription factors modulating hBD-1 promotor activity. Significant increase in luciferase activity (**A**) in A549 cells with co-transfection of the hBD-1-luciferase plasmid construct with interferon regulatory factor (IRF)-7 and PU.1 expression vectors and significant decrease in luciferase (Luc2) activity with co-transfection of an IRF-5 expression vector compared with the construct alone (*p*-value < 0.05). (**B**) Significant increase in luciferase activity over time with herpes simplex virus-1 (HSV-1) infection of OKF6/TERT cells transfected with the hBD-1 promoter/luciferase reporter construct (*p*-value < 0.05). Statistical analysis done with analysis of variance (ANOVA) and Tukey’s test for multiple comparisons and significant increases are indicated by one asterisk and the significant decreases are indicated by double asterisks. Expression vectors were provided by Betsy Barnes (Feinstein Institute, Manhasset, NY, USA). Transfections and analyses were carried out as described elsewhere [26].

To gain insight as to what transcription factors regulate hBD-1 gene expression, we analyzed the upstream flanking region of hBD-1 for putative transcription factor binding sites using MacVector (MacVector, Inc., Apex, NC, USA) and MatInspector (Genomatix, GmbH, Munich, Germany) sequence analysis programs. The sequence shown in Figure 1A indicates several potential sites for transcription factor binding. Most interesting is the placement of a composite site for the cooperative binding of PU.1/spleen focus forming virus (SFFV) proviral integration oncogene (SPI1) with an interferon regulatory factor (IRF) site. This is similar to what is seen in the enhancer region of IL-1β. The PU.1, and some IRF and signal transducer and activator of transcription 1 (STAT1) sequences, are conserved between mouse and human, suggesting that regulation of these BD-1 homologues is similar. Analysis of the upstream flanking region of the hBD-1 gene suggests that it is regulated by IRFs. To delve further into the regulation of hBD-1, we utilized a luciferase reporter system in A549 cells co-transfected with expression vectors containing IRF3, IRF5, IRF7 or PU.1. In Figure 2A, our results showed that IRF7 and PU.1 were the only transcription factors that increased activation of the hBD-1 promoter and that IRF5 decreased hBD-1 promotor activity. IRF3 had no effect on hBD-1 promoter activity. In addition, in Figure 2B, when OKF6/TERT cells were transfected with the hBD-1 promoter-luciferase reporter expression vector, then infected with HSV-1, luminescence increased significantly and exponentially at 4, 6 and 8 h after infection [24].

These above results suggest that HSV-1 induction of hBD-1 in PDC occurs independently of IFN-α at the receptor level but that transcription factors common to IFN-α gene regulation are involved in the regulation of hBD-1 gene expression. This is in stark contrast to the effect of LPS incubation of airway epithelium, in which hBD-1 is not induced but remains at steady-state levels [9]. LPS induction of hBD-2 is mediated via an NF-κB signaling pathway and is initiated via a TLR-4 receptor in epithelial cells [19]. LPS does not stimulate PDC to make hBD-1 or hBD-2, indicating that the NF-κB pathway is not directly active in inducing these two β-defensins in PDC. However, LPS stimulation is not ineffective, for LPS activation of the NF-κB pathway may indirectly affect hBD-1 production by HSV-1. When HSV-1 was incubated with PDC in the presence of LPS, IFN-α production induced by the virus was enhanced due to the increase in TLR9 and IRF-7 [27]. It is possible that LPS could also enhance hBD-1 production by HSV-1 as well, since hBD-1 production appears to be mediated via IRF-7.

The variability in hBD-1 induction in response to virus is likely to be at least partially influenced by genetic polymorphisms in the hBD-1 locus. HBD-1 regulation is affected by SNPs in the promoter region and is associated with virus susceptibility. Polymorphisms in the hBD-1 promoter region have been associated with children and adults with HIV [28,29,30,31]. However, SNPs have not only been associated with viral infection susceptibility. Polymorphisms have also been associated with chronic obstructive pulmonary disease [32] and *Candida* carriage [33].

## 6. In Vivo Induction of hBD-1 by Virus in Humans

Is in vitro induction of hBD-1 relevant in viral disease? What about in vivo induction of hBD-1? The direct antiviral activity of hBD-1 was shown to be weak at best against HSV-1 [23], suggesting that other mechanisms are in play. The first study to show that virus induced hBD-1 in vivo was just published [25]. The results showed the first characterization of induction of hBD-1 (or any hBD) during acute and chronic viral infection in humans. The results depicted hBD-1 mRNA and peptide induction in conventional monocytes (CD14^+^CD16^−^) in acute HIV-1 (<3 months) infection over constitutive production in unstimulated monocytes. HBD-2 and hBD-3 were not increased in the PBMC of these acutely infected patients. As the infection progressed to the chronic stages, hBD-1 production decreased back to constitutive levels in PBMC and monocytes. The study provided important insight into the in vivo kinetics of hBD expression, the mechanism of hBD-1 induction by HIV-1, and the role of hBDs in the early innate response to HIV-1 acute infection. In this study, direct hBD-1 activity against HIV-1 was weak in vitro also. In vivo, there was no correlation between viral load and hBD-1 levels in the HIV-1 patients. They also assessed punch biopsies containing mostly epithelial cells in the transverse colon, terminal ileum and duodenum of HIV patients. Although hBD-1 levels were much higher in the intestinal epithelial cells, no increase of hBD-1 occurred in patients with acute infection as it did in their monocytes gated from PBMC compared with patients having chronic disease (HIV-1 chronic progressors and HIV-1 viremic controllers) or uninfected HIV negative control subjects. However, transcription of hBD-1 mRNA in the gut was 10–100 times higher compared with PBMCs. These results support the hypothesis that hBD-1 does not affect viral titers directly following viral infection, but plays a role in directing the local early innate immune response to the virus. These early innate immune responses may include attracting DC and T-lymphocytes to the area, as well as adjuvant effects enhancing tissue macrophage responses to the virus itself, and may influence its own production in monocytes and that of IFN-α in PDC. It has already been shown that recombinant hBD-1 and recombinant hBD-2 have chemoattractive properties for immature DC and memory T-cells via binding to the C–C motif chemokine receptor 6 (CCR6) on these cells [34]. It has also been shown that gp120-hBD-2 DNA constructs can enhance antibody responses to the HIV envelope glycoprotein, gp120, in mice, suggesting that hBD-1 and hBD-2 may influence the cytotoxic lymphocyte response to virus at mucosal surfaces [35]. It is possible that variances in the type of early innate immune response of β-defensins may occur with different types of viruses. More research is needed to determine the exact influence of hBD-1 on early viral innate immune responses of HIV and other viral infections. The viruses and viral ligands that interact with β-defensins have been summarized in several recent review articles [2,36,37,38,39]. However, hBD-1 has not been extensively studied.

Other studies of hBD-1 gene expression increase in viral infection in humans have been noted. Dengue virus increased hBD-1 mRNA early in infection, detected in RNA extracted from whole blood samples [40]. Human papilloma virus also increased hBD-1, when active lesions were compared with unaffected normal tissue in the same patient [41]. Rhinovirus has also been found to induce hBD-1 mRNA in nasal epithelial brushings from patients with acute cold, but patients with cystic fibrosis did not have this increase, suggesting that hBD-1 regulation is influenced by the disease-state of the host and other host factors [42].

## 7. In Vivo Induction of hBD-1 in Animal Models of Viral Infection

Animal models of viral infection also seem to support the idea that hBD-1 is induced by virus and is functioning by modulation of early innate immune responses. In sheep, sBD-1 mRNA was increased in lung homogenates as early as three days following infection with parainfluenza virus 3; sBD-1 has a tissue distribution and constitutive production in epithelial cells similar to hBD-1 [43]. In mice, other studies have shown viral induction of the hBD-1 mouse homologue, mBD-1 [44,45]. Figure 1B illustrates a comparison of the types of regulatory sequences between humans and mice. In a mouse model using influenza A/Hong Kong/8/68 (H3N2) virus, four mouse β-defensins were expressed in the lungs, trachea and nasosinuses during the first six days of infection [46]. mBD-1 mRNA was increased over uninfected baseline in limited, consistent amounts in the nasosinus and trachea but in the lung, mBD-1 gene expression increased greatly over control early in the course of infection—day one and day two after infection—and then declined to less than a one-fold increase over control on days three and six. Ryan et al. showed that when mBD-1 was deleted, mice infected with influenza virus showed an increased morbidity and mortality, along with increased lung pathology over wild type mice infected with mouse-adapted influenza A/Hong Kong/1968 (H3N2) virus. Deletion of mBD-1 resulted in an earlier and greater number influx of inflammatory cells into the lung, increased perivascular edema and enhanced diffuse alveolar hemorrhage compared with infected wild type C57Bl/6 mice [23]. The lung virus titers were equal 24 h after infection in wild type and mBD-1^−/−^ mice, suggesting that mBD-1 played a role in inhibiting the strong innate immune and inflammatory response to the virus, rather than by inhibiting virus titers.

## 8. Viral Innate Immune Escape

Paradoxically, virus infection in vitro can suppress hBD-1 in both gingival and airway epithelial cells. This was shown in our lab using HSV-1 infection of OKF6/TERT2 cells [23]. The virus was tagged with a green fluorescent protein (GFP)-cytomegalovirus (CMV) promotor such that when the virus replicated, infected cells appeared green. There was an inverse correlation with hBD-1 mRNA, detected with quantitative (q)RT-PCR, and viral infection. GFP began appearing about 8 h after infection and significant suppression of hBD-1 mRNA began at 4 h and maximized at 8 h after infection. Only live HSV-1 was able to suppress hBD-1; neither UV-inactivated HSV-1 nor the TLR9 ligand, CpG DNA, have any effect [23].

To support this hypothesis, influenza A virus PR8 (H1N1) also suppressed steady-state levels of hBD-1 in primary, differentiated and undifferentiated NHBE cells [23]. These results hint that there may be a mechanism to suppress early innate immune responses of the cell upon viral entry. However, four in vivo or in situ studies have shown that hBD-1 is increased upon viral infection of the host in humans, mice and sheep and the source of hBD-1 may be in the monocytic cell response or gut epithelial cells. One of the viral infections in the mouse model was influenza and induced hBD-1 in lungs, but the cells were not characterized [46]. In our study, when epithelial cells were stimulated with polyinocinic:polycytidylic acid (poly I:C), a ligand simulating double-stranded RNA virus stimulation via TLR3, hBD-1 increased three-fold over control. Thus, specific virus type, active state of the virus (live or attenuated), and cell type can influence the direction of hBD-1 modulation [23]. The mechanism of specific viral components and their effect on hBD-1 modulation has not been thoroughly investigated.

## 9. Conclusions

Modulation of hBD-1 gene expression in viral infection occurs in various scenarios and is influenced by host environmental factors. There is evidence that this modulation could influence the outcome of viral disease. In addition, genetic mutations in the hBD-1 promoter have been shown to affect susceptibility to viral disease; and perhaps the ability of monocytic cells to respond to virus with hBD-1, in addition to IFN-α, may affect host cell susceptibility to infection by virus. The research in this area is in its infancy. More research to understand the regulation of gene expression and protein translation of endogenous antimicrobial peptides, particularly hBD-1, will lead to better therapeutic options for controlling virus infection, immunity and pathogenesis.
